# Supplementary figures and images for: Protective effect of sucrose esters from cape gooseberry (Physalis peruviana L.) in TNBS-induced colitis
Source: PLoS One. 2024 Mar 21;19(3):e0299687. doi: 10.1371/journal.pone.0299687 (PMC10957089; doi:10.1371/journal.pone.0299687)

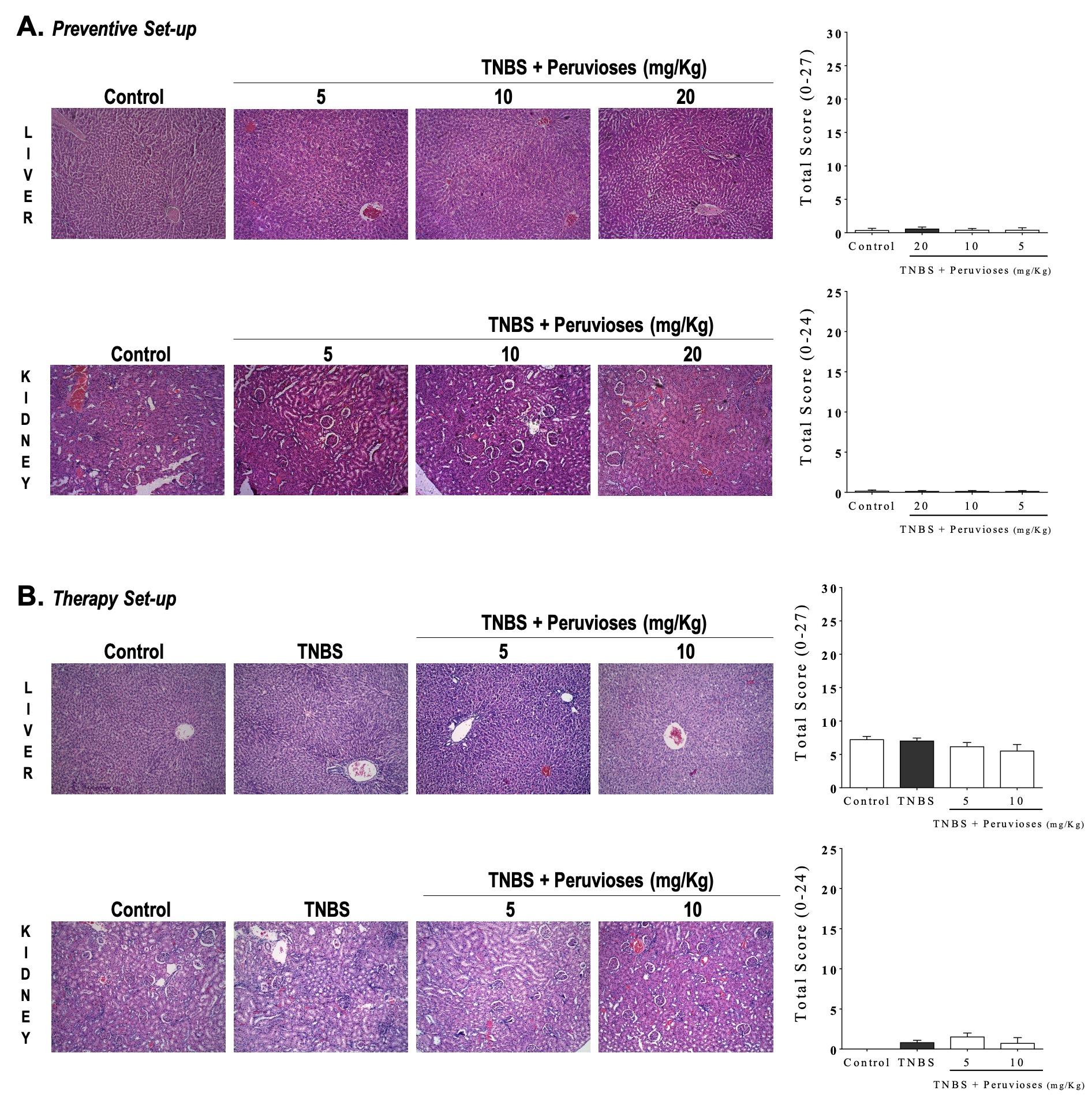

Supplement: S1 Fig — Treatment with Peruvioses A and B mixture (0–20 mg/Kg/day, ip) isolated from Physalis peruviana calyces did not produce an effect on the histological structure of liver and kidneys of TNBS-treated rats in the preventive (A) or therapy (B) set-up. Micrographs are representative of the histological section of organs stained with hematoxylin and eosin from at least six different animals. Magnifications 10X. Scores were assigned by a blinded pathologist according to the parameters established in S1Table, Supplementary Information. Each value represents the mean ± SEM. (TIF) [file pone.0299687.s001.tif]

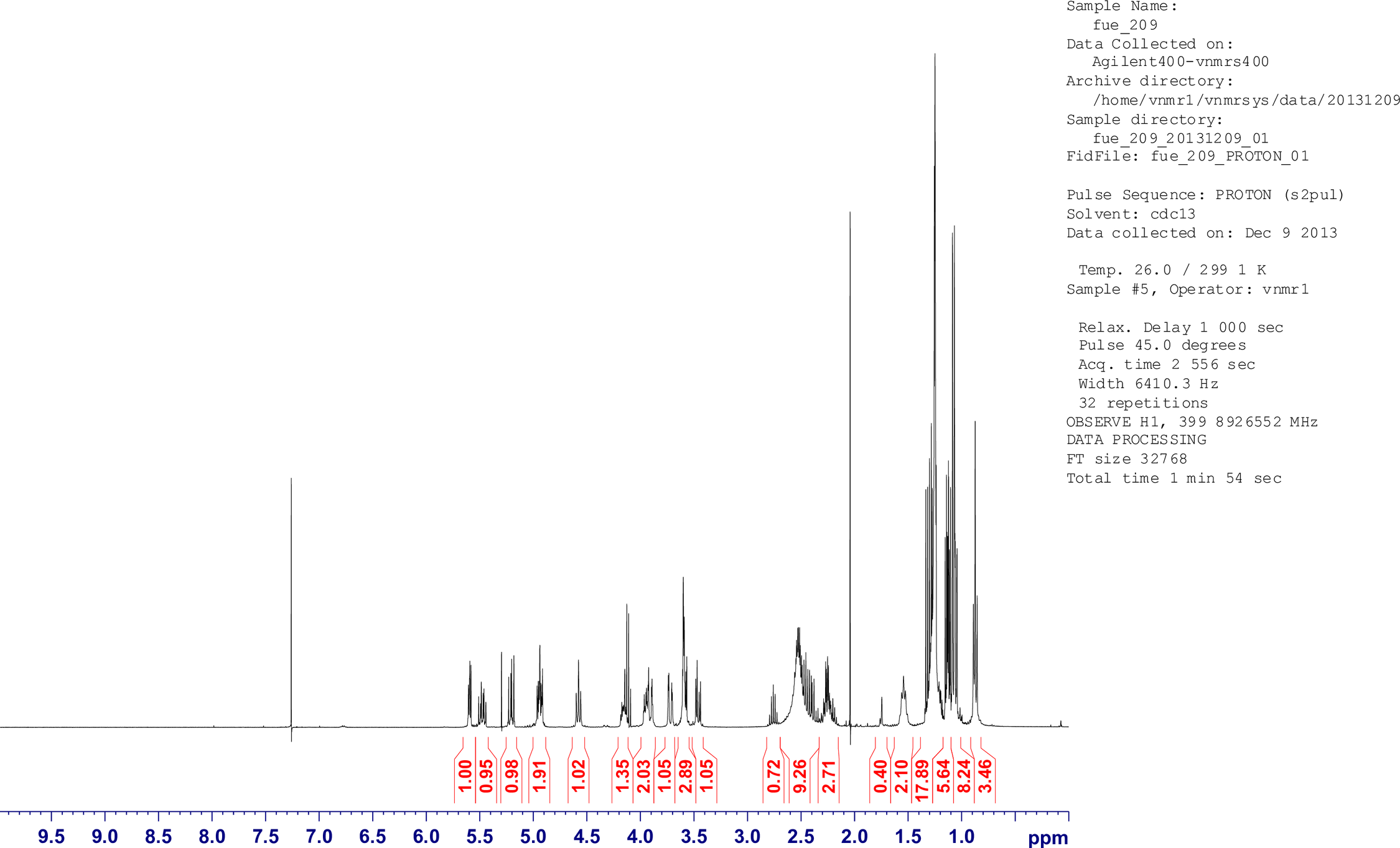

Supplement: S2 Fig — (TIF) [file pone.0299687.s002.tif]

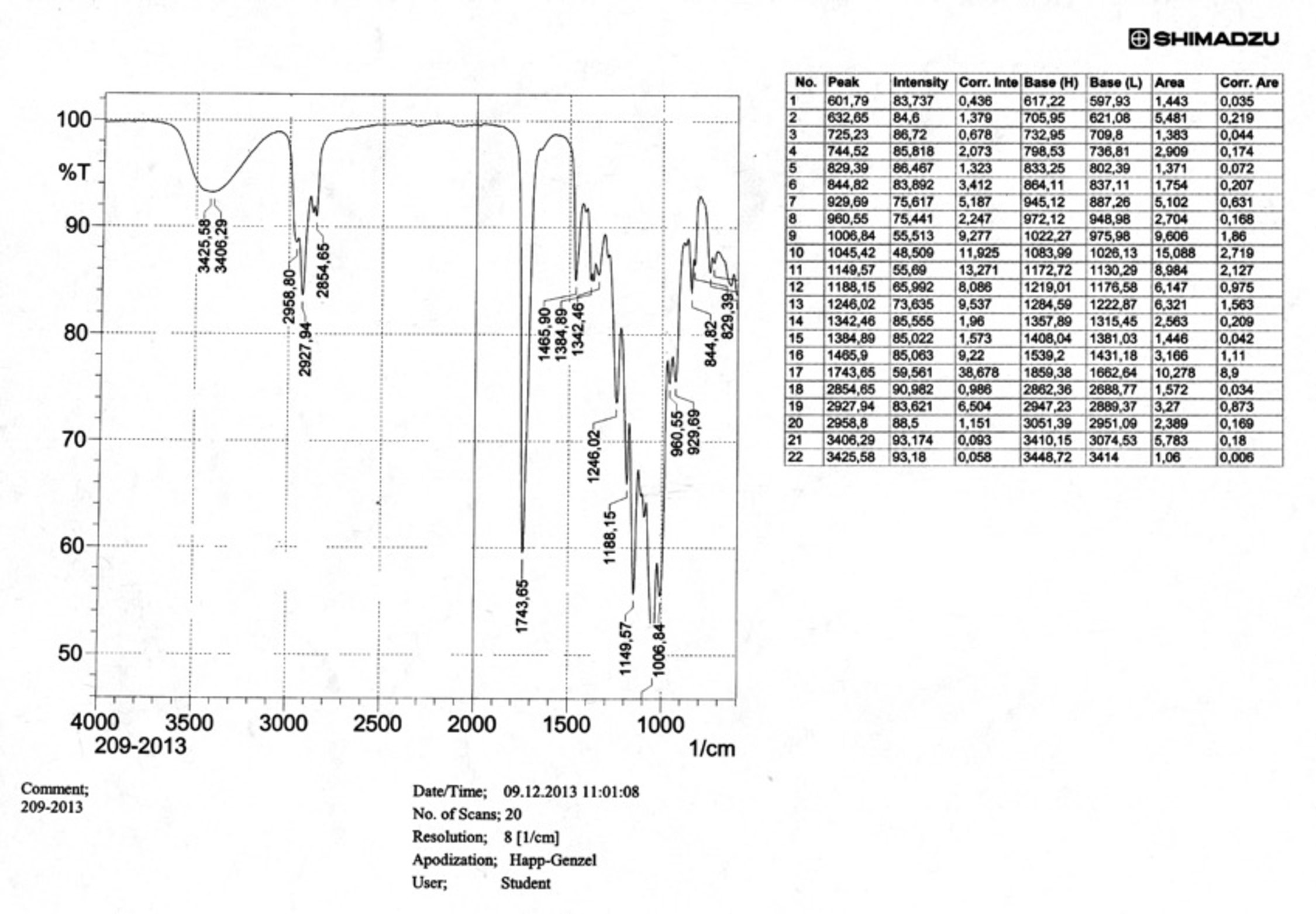

Supplement: S3 Fig — (TIF) [file pone.0299687.s003.tif]

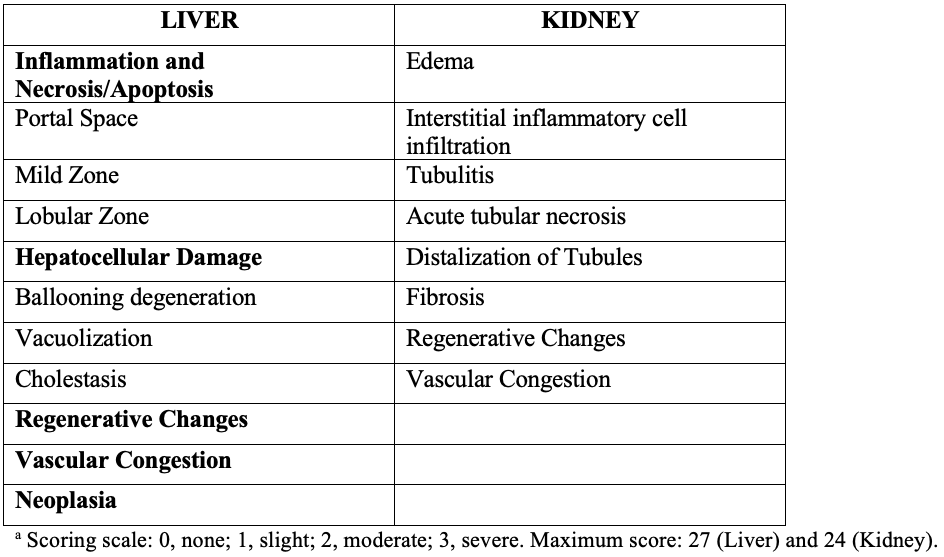

Supplement: S1 Table — (TIF) [file pone.0299687.s004.tif]

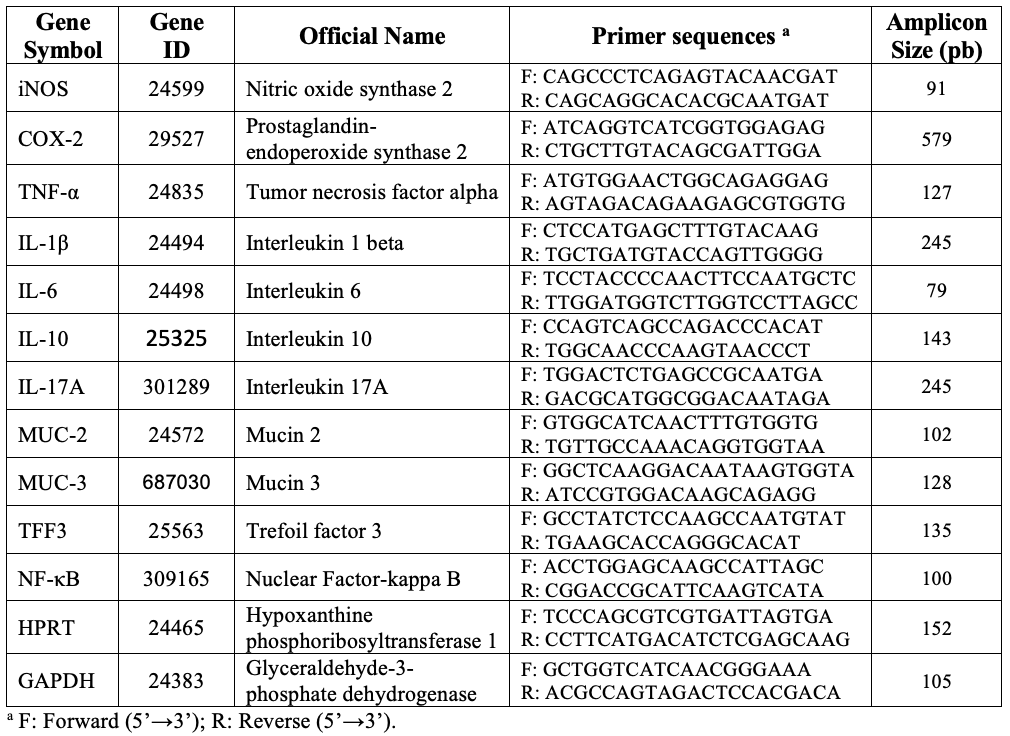

Supplement: S2 Table — (TIF) [file pone.0299687.s005.tif]
