# Supplementary material for: Protective effect of sucrose esters from cape gooseberry (Physalis peruviana L.) in TNBS-induced colitis
Source: PLoS One. 2024 Mar 21;19(3):e0299687. doi: 10.1371/journal.pone.0299687 (PMC10957089; doi:10.1371/journal.pone.0299687)
Supplement: Attachment — Submitted filename: Response to Editorial Board with annexes.pdf [file pone.0299687.s006.pdf]

Cartagena, October 6th 2023

Doctor
Emily Chenette
Editor-in-chief
Plos-One

Appreciated Dra. Chenette, in response to the request on our submission **PONE-D-23-23074**, "***Protective effect of sucrose esters from Cape gooseberry (Physalis peruviana L.) in TNBS-induced colitis***" by Yanet Ocampo et al., we attached the required documents:

Annex 1. Completed PLOS ONE Humane Endpoints Checklist.

Annex 2. English translation of the original ethics approval document.

Please do not hesitate to contact me if you have any questions.

Sincerely,

Prof. LUIS FRANCO OSPINA, Ph.D.
Corresponding author
Biological Evaluation Group Director
Universidad de Cartagena - Colombia.

Faculty of Pharmaceutical Sciences – Biological Evaluation Group
Campus de Zaragocilla, Área de la Salud
Teléfonos: 57-5-6699771
email: lfrancoo@unicartagena.edu.co
web: www.unicartagena.edu.co
Cartagena de Indias, D.T. y C. - Colombia

PLOS ONE Humane Endpoints Checklist

PLOS ONE manuscript number: _ PONE-D-23-23074___

Complete the following if your study design includes death of a regulated animal as a likely outcome or planned experimental endpoint. Please also include all information in the Methods section of your manuscript.

ITEM 1. Describe whether humane endpoints* were used for all animals involved in the study.

	Recommendation	Section/Paragraph
If humane endpoints were used, report the following:*		
1	The specific criteria used to determine when animals should be euthanized	*2. Material and methods 2.3. Animals / paragraph 1. line 151-155*
2	Once animals reached endpoint criteria, the amount of time elapsed before euthanasia	*2. Material and methods 2.3. Animals / paragraph 1. line 151-155*
3	Whether any animals died before meeting criteria for euthanasia	*No animals died before meeting the criteria for euthanasia*
If humane endpoints were **not** used, report the following:*		
1	A scientific and ethical justification for the study design, including the reasons why humane endpoints could not be used, and discussion of alternatives that were considered but could not be used	*This criterion does not apply*
2	Whether the institutional animal ethics committee specifically reviewed and approved the anticipated mortality in the study design	*This criterion does not apply*

ITEM 2. Include the following details of the study design and outcomes.

	Recommendation	Section/Paragraph
1	The duration of the experiment	*2. Material and methods 2.5. Induction of TNBS colitis.*

		line 180-185
2	The numbers of animals used, euthanized, and found dead (if any); the cause of death for all animals	*The number of animals used in each test is specified in the legend of the corresponding figure.*<br>*No animals were found dead or euthanized during the experiments.*
3	How frequently animal health and behavior were monitored	*2. Material and methods*<br>*2.5. Induction of TNBS colitis.*<br>*line 186-187.*
4	All animal welfare considerations taken, including efforts to minimize suffering and distress, use of analgesics or anaesthetics, or special housing conditions.	*2. Material and methods*<br>*2.3. Animals /*<br>*line 141-155*<br>*2.5. Induction of TNBS colitis.*<br>*line 170-178.*
5	Any special training in animal care or handling provided for research staff	*The scientific personnel involved in animal testing were trained and experienced in the handling and care of experimental animals.*

***Definition of a humane endpoint**

A humane endpoint is an experimental endpoint at which animals are euthanized when they display early markers associated with death or poor prognosis of quality of life, or specific signs of severe suffering or distress. Humane endpoints are used as an alternative to allowing such conditions to continue or progress to death following the experimental intervention ("death as an endpoint"), or only euthanizing animals at the end of an experiment. Before a study begins, researchers define the practical observations or measurements that will be used during the study to recognize a humane endpoint, based on anticipated clinical, physiological, and behavioral signs. These may include, for instance, body temperature or weight changes, tumor size or appearance, abnormal behaviors, pathological changes, ruffled fur, reduced mobility, body posture, or expression of specific body fluid markers. Please see the NC3Rs guidelines for more information.

ARRIVE Guidelines

PLOS ONE encourages authors to follow the Animal Research: Reporting of In Vivo Experiments (ARRIVE) guidelines for all submissions describing laboratory-based animal research and to upload a completed ARRIVE Guidelines Checklist to be published as supporting information.

THE PRESIDENT OF THE RESEARCH ETHICS COMMITTEE OF THE UNIVERSITY OF CARTAGENA

CERTIFIES THAT:

The research project entitled "IDENTIFICATION OF MOLECULAR TARGETS INVOLVED IN THE ANTI-INFLAMMATORY AND IMMUNOMODULATORY EFFECT OF ISOLATED COMPOUNDS FROM PHYSALIS PERUVIANA L., USING AN ULCEROUS COLITIS MODEL", presented by LUIS ALBERTO FRANCO OSPINA, Professor of the Pharmaceutical Science Faculty of the University of Cartagena has no ethical impediments according to the Resolution 8430 of 1993 of Ministry of Health and the ethics regulation of the University of Cartagena, as recorded in the minute No. 23 of Research Ethical Committee of October, 14th of 2010.

For the record, this document is signed in Cartagena city on October, fourteenth (14) of the year two thousand ten (2010).

ALVARO OLIVERA DIAZ, MD
President
